# Removal torque pattern of a combined cone and octalobule index implant-abutment connection at different cyclic loading: an in-vitro experimental study

**DOI:** 10.1186/s40729-018-0154-2

**Published:** 2019-01-14

**Authors:** Kanyarin Benjaboonyazit, Pisaisit Chaijareenont, Pathawee Khongkhunthian

**Affiliations:** 1 Center of Excellence for Dental Implantology, Faculty of Dentistry, Chiang Mai University, Chiang Mai, Thailand; 2 Department of Prosthodontics, Faculty of Dentistry, Chiang Mai University, Chiang Mai, Thailand; 3 Center of Excellence for Dental Implantology, Faculty of Dentistry, Chiang Mai University, Suthep, A. Muang, Chiang Mai, 50200 Thailand

**Keywords:** Abutment screw, Removal torque, Octatorx, Cyclic loading, ISO 14801

## Abstract

**Background:**

Despite the high survival rate of dental implants, screw loosening is frequently reported. Screw loosening can cause a misfit of the implant-abutment connection leading to peri-implantitis or abutment screw fracture. Therefore, studies about related factors and mechanism of screw loosening are needed. The aim of this study was to evaluate the decreasing pattern of removal torque values (RTVs) of a combined cone and octalobule index implant-abutment connection under different numbers of mechanical loading cycles.

**Materials and methods:**

The study was performed in accordance with ISO 14801:2007. Eighty-four implants with the combined cone and octalobule index implant-abutment connection (PW Plus dental implant system, PW Plus Company) were used. All abutment screws were tightened 30 N cm twice with a 10-min interval. The control group was without cyclic loading and the experimental groups underwent different numbers of loading cycles until 2,000,000 cycles. Then, the abutment screws of all samples were untightened to measure the RTVs. The data were analyzed using ANOVA and Tukey’s HSD test.

**Results:**

The RTVs of the control group decreased 7.78% compared to the insertion torque. All experimental groups from 50,000 to 2,000,000 cycles showed significant decreases in RTVs compared to the control group (*P* < 0.05). RTVs in the group of 50,000 cycles to 1,800,000 cycles did not change significantly, but there was a significant reduction of RTVs in the group of 2,000,000 cycles when compared to the group of 50,000 cycles (*P* < 0.05).

**Conclusions:**

According to the setting condition for the fatigue test complied to ISO 14801:2007, the RTVs of the combined cone and octalobule index implant-abutment connection reduced significantly after 50,000 cycles and did not change significantly until 2,000,000 cycles.

## Background

**Fig. 1:**
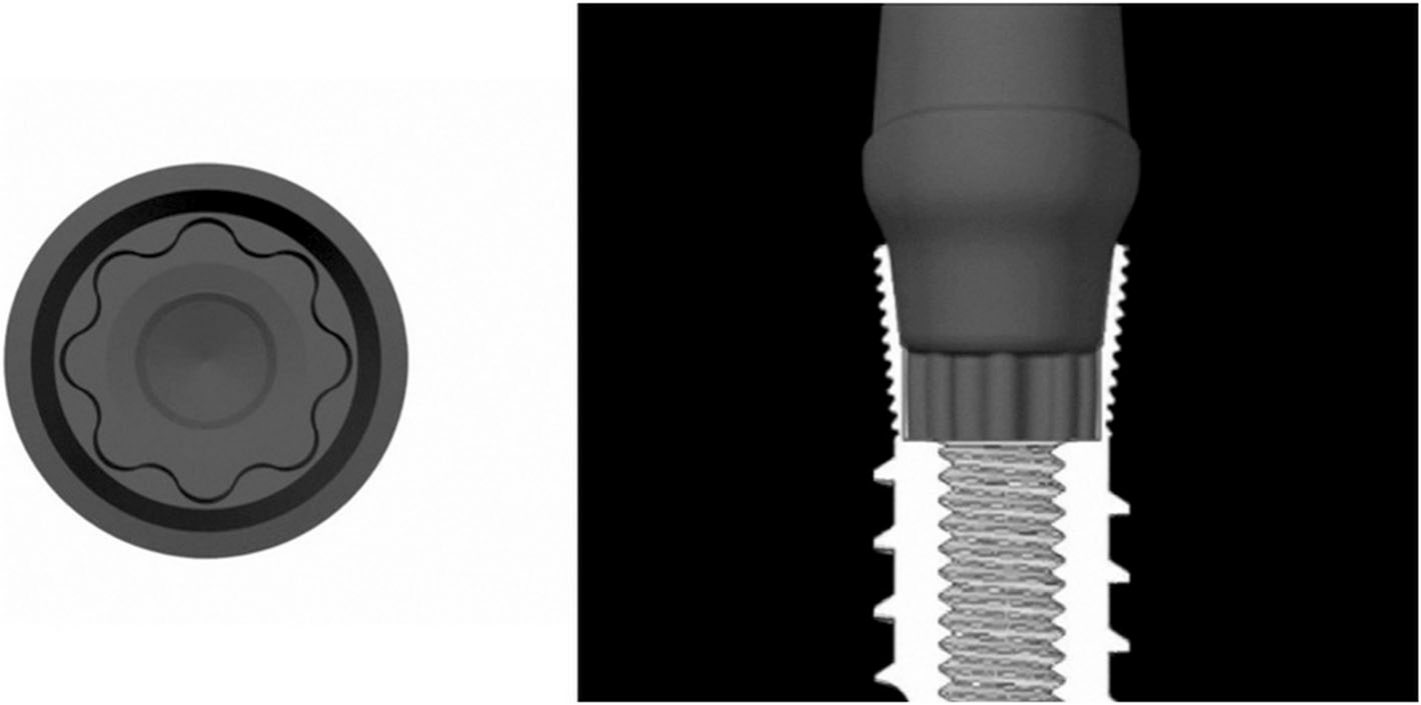
Cone connection combined with octalobular index (Octatorx)

Dental implant placement has shown high survival and success rates [1, 2]. According to one study, the overall 5-year prosthetic survival rate for implant treatment has increased from 93.5 to 97.1% during the past 10 years [1]. Despite the high survival rate of implants, technical complications, such as screw loosening, are frequently reported [1–3]. The 5-year complication rate for screw loosening in studies after the year 2000 is 8.7%. Screw loosening can cause a misfit of the implant-abutment connection [4] which may lead to a gap, increasing bacterial accumulation, and possible peri-implant tissue inflammation. Moreover, using an implant prosthesis with a loose screw can result in abutment screw fracture [5]. One of the key factors in the success of dental implant treatment is the implant-abutment connection [6]. One such connection is the internal cone connection which has been reported to improve the biomechanical properties of implant-abutment assemblies [7]. If the contacting angle of the cone connection is 2° to 8°, the connection is called a Morse taper connection [8]. The Octatorx-cone connection is a combination of a Morse taper connection with a 6° tapered angle and star-shaped “octalobules” indices with eight rounded points [9] (Fig. 1). This Octatorx-cone connection provides an anti-rotational characteristic and surface frictional resistance at the implant-abutment interface, which may prevent micromovement and screw loosening during function.

The stability of the implant-abutment connection comes from both the screw function and the frictional resistance between the conical, contacting metallic surfaces of the connection [10]. An abutment screw provides stability via a clamping force [11, 12]. When rotational torque is applied to the screw, the screw elongates and causes stress on the stem and threads. After that, elastic recovery of the screw occurs, the stem and threads of the screw are in tension and a clamping force is created, pulling the abutment and the implant together. This clamping force is parallel to the axis of the implant and is also known as preload. The preload value is directly proportional to screw elongation. The more screw elongation remains after elastic recovery, the greater is the preload value.

The occlusal force does not load only on the screw. The internal conical connection interface also helps to transfer and distribute the loading force to the implant [13]. The axial compressive component of occlusal force during oral function causes axial displacement of the abutment to the implant connection, increasing the frictional resistance and screw joint stability of the dental implant [7, 8]. Three factors that may lead to the axial displacement of the implant-abutment connection are machining tolerance, the wedge effect, and the settling effect. Firstly, machining tolerance is a dimensional variation characteristic of machined components. The precision of every implant component varies during manufacturing due to machining tolerance [14]. Secondly, the wedge effect occurs when tightening torque or loading force is applied to the abutment [15]. The abutment acts as a wedge transferring the axial force directly to the implant. Lastly, the settling effect occurs when rough spots on the contacting surfaces of the connection are flattened under load [16]. It causes the two surfaces to come closer together and leads to axial displacement. This axial displacement causes the length of the abutment screw to shorten, diminishing the screw preload [17]. The settling effect is the main cause of screw loosening. When the abutment is fastened to the implant body with the abutment screw, a settling phenomenon occurs in which the implant body and the abutment are deformed. Even if fastened at 30 N cm, depending on the implant system, the loosening torque becomes smaller than the 7 to 10% fastening torque. After 5 min from this point, it is necessary to loosen it twice [18]. The degree of settling depends on the surface roughness, surface hardness, and magnitude of the tightening torque and of the occlusal loading force [16]. On the one hand, the settling effect [17] causes the axial displacement leading to the decrease of the screw preload. On the other hand, the axial displacement causes the cone connection surface adaptation providing frictional resistance and screw-joint stability [7, 8].

Bickford has explained how the screw is loosened in two stages [19]. Firstly, an external force causes micromovement and slipping between the abutment screw thread and the implant, decreasing the screw preload. Secondly, if the preload value falls below a critical level, and the external force exceeds the joint separating force, then screw loosening occurs. Consequently, the higher the preload value, the greater is the resistance to screw loosening.

Many factors affect the screw preload, such as the coefficient of friction between the surface, the elastic modulus of the screw, the geometry of the screw and of the connection, the fit of the components, insertion torque, the presence of lubricant, and occlusal overload [12]. The optimal screw preload should induce a stress in the joint of about 60–75% of the yield strength of the material [20]. There are many methods to measure screw preload [21], removal torque measurement being the most common [22]. The removal torque is the rotational force used to loosen the screw. The greater the preload obtained, the higher is the removal torque value.

**Fig. 2:**
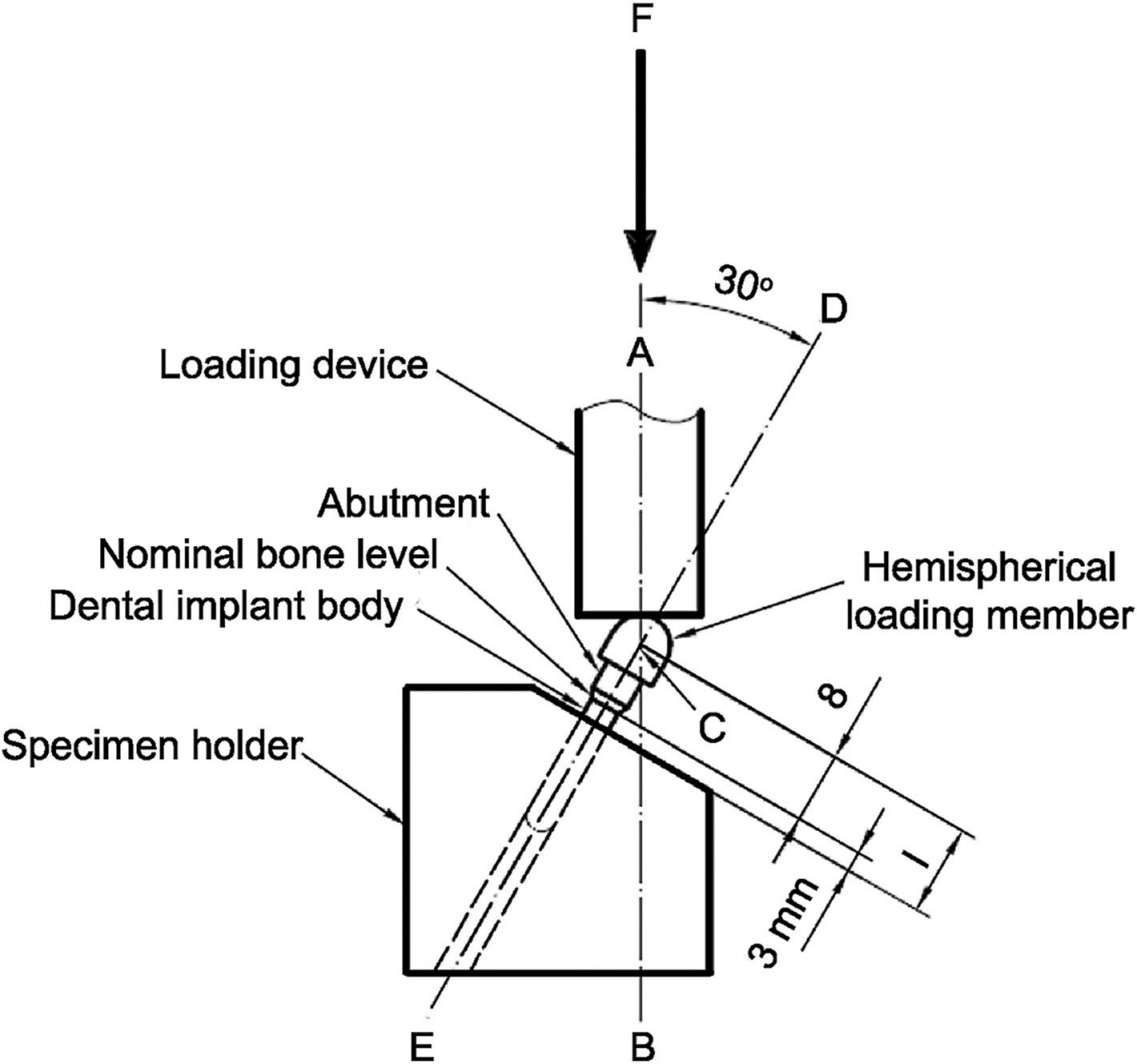
Schematic of test setup according to The ISO 14801 recommendations

The literature demonstrates that humans undergo about 800,000 chewing cycles in a year [23], with the frequency of cycles ranging from 1 to 19 Hz [24]. Lundeen and Gibbs reported that the average occlusal force for fixed prostheses supported by implants is 250 N [25]. Different methods of cyclic testing have been used to simulate clinical oral function. The ISO 14801:2007 recommendations were designed for single, endosteal, transmucosal dental implants tested under “worst case” applications [26]. The implant samples protrude from a supporting resin by 3 mm, simulating the worst situation of 3 mm of vertical bone resorption around the implant. The implant samples are angled at 30° to the vertical and are stressed by vertical and oblique loads (Fig. 2).

In most studies, the removal torque values (RTVs) decreased after cyclic loading [27, 28], but in some studies, higher RTVs were reported [29, 30]. There are many independent variables that may influence the screw preload as mentioned above [12]. Paepoemsin et al. [31] evaluated the removal torque of the tapered screws and flat-head screws of an implant system and found that the RTVs reduced significantly after 1,000,000 cycles of loading. Few studies have truly focused on the effect of functional loading duration on preload maintenance of the screw. How the screw preload may change under different functional loading times is still unknown. The significant reduction of the RTVs and the patterns after cyclic loading have also not been investigated. Therefore, the purpose of the study was to investigate the RTVs’ decreasing pattern after different cyclic loading. The null hypothesis was that the RTVs are not significantly reduced after different cyclic loading.

## Materials and methods

**Fig. 3:**
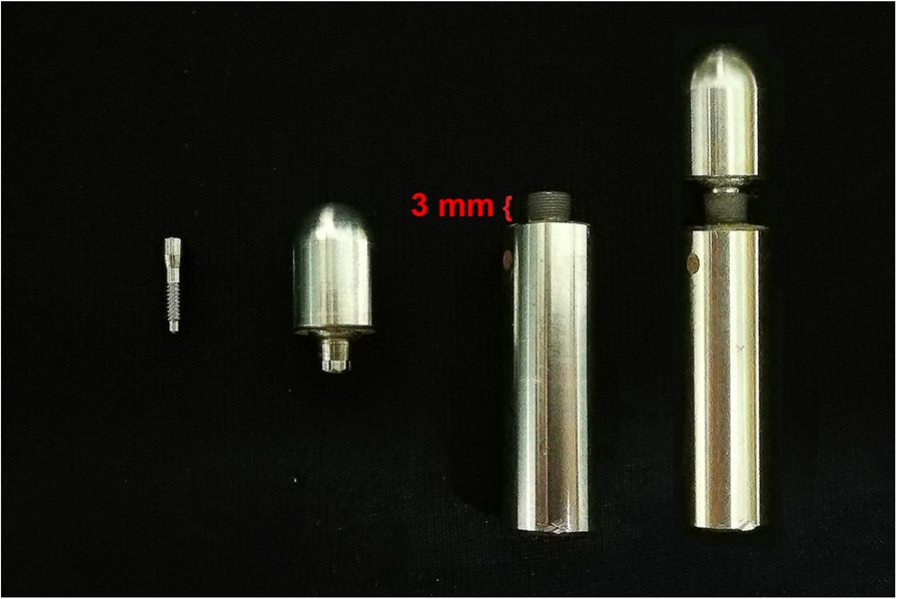
Implant assembly embedded in resin block

**Fig. 4:**
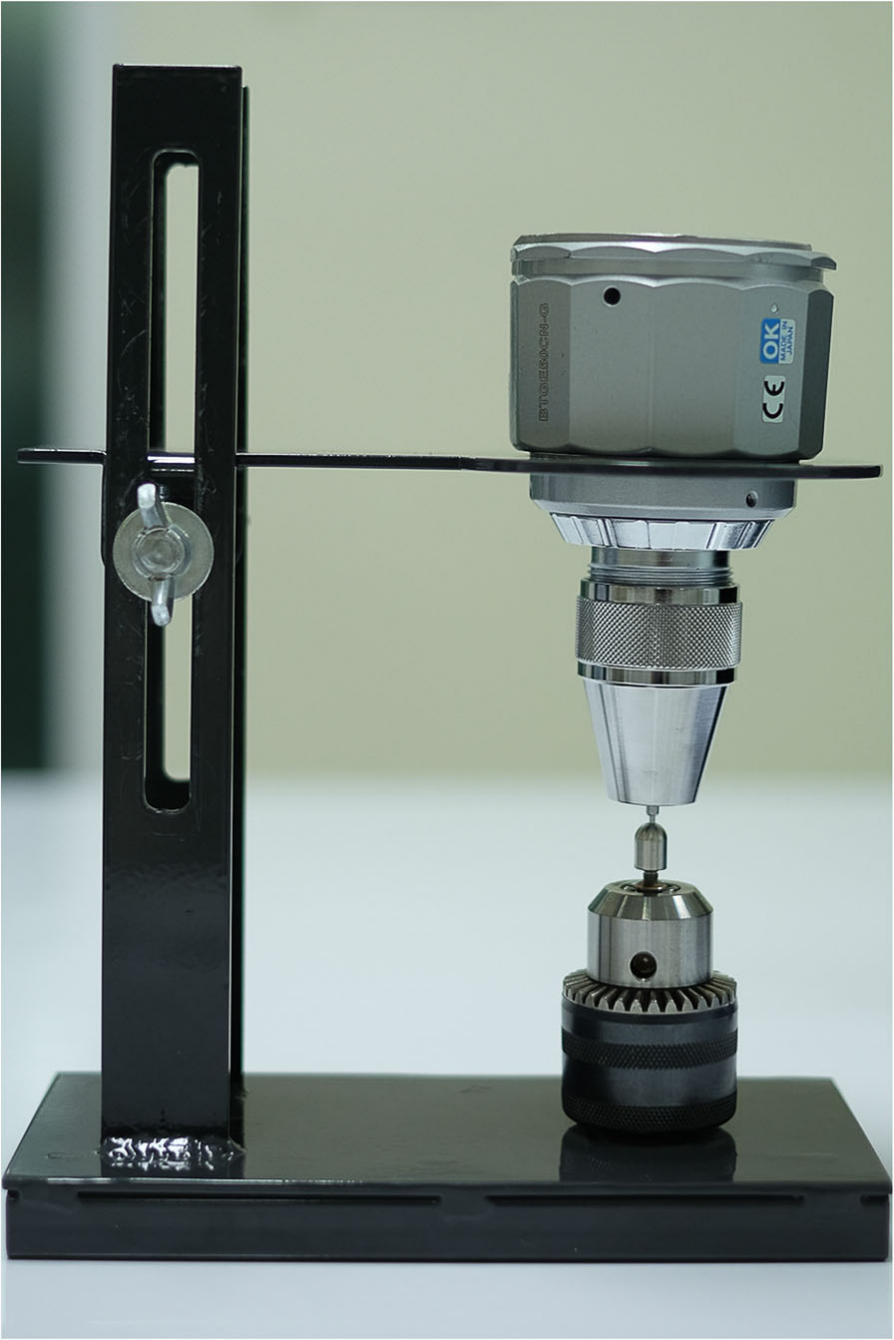
Tightening the abutment screw and measuring the RTVs

**Fig. 5:**
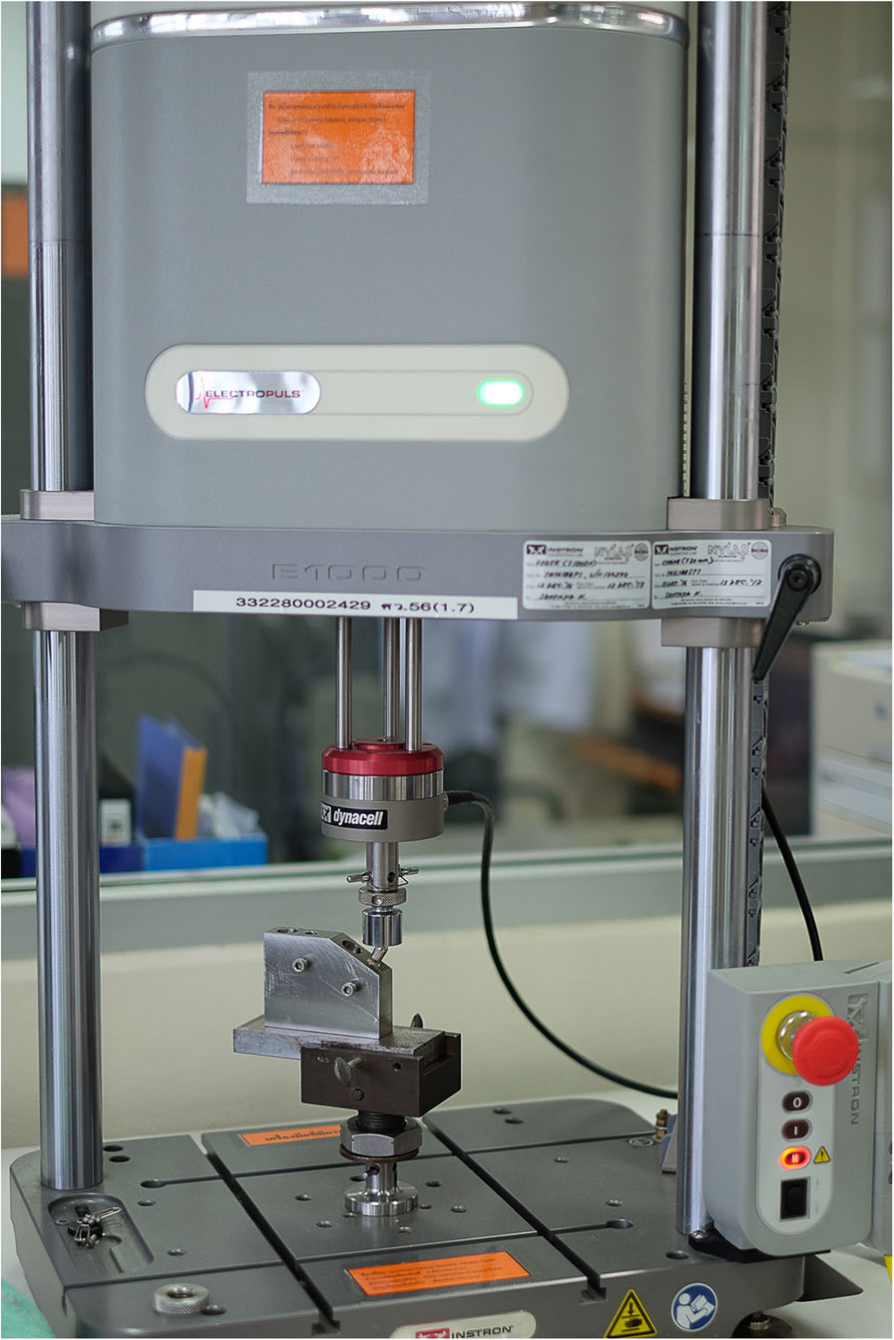
Specimen mounted in a 30°-angled steel holder in ElectroPuls E1000 dynamic testing machine

**Fig. 6:**
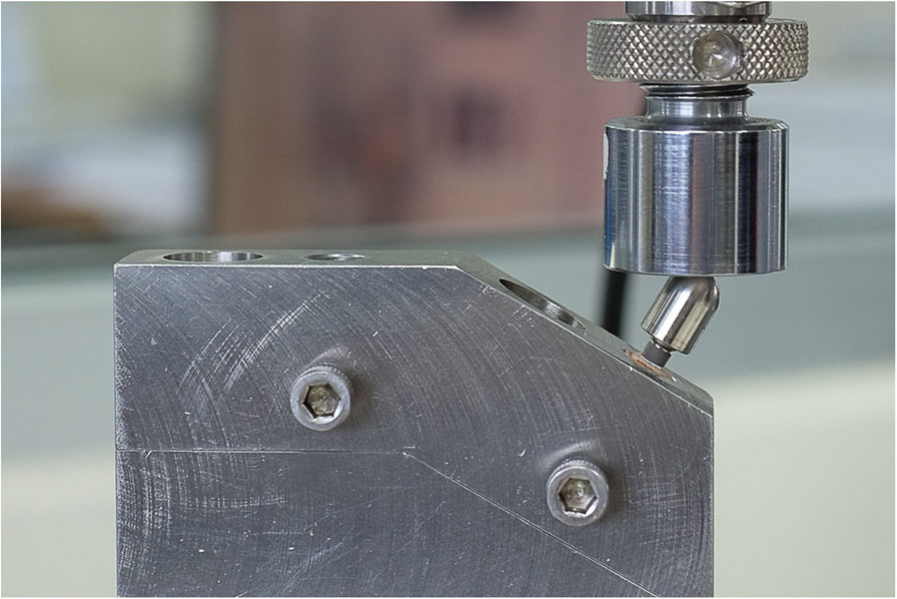
Specimen mounted in a 30°-angled steel holder

This study was conducted in accordance to the international standard fatigue test (ISO 14801:2007) for endosseous dental implants in vitro (Fig. 2). Eighty-four implants (PW Plus implant system, PW Plus Company) were prepared, which had a diameter of 3.75 mm and a length of 10 mm, with the Octatorx-cone implant-abutment connection. All were embedded in individual epoxy resin blocks (Chockfast Orange Resin, Shannon Industrial Estate, Co. Clare, Ireland). The platforms of the implants were 3 mm above the level of the upper border of the resin block. The straight abutment of the implant sample was attached to a hemispherical metal cap, which was relined with Chockfast resin on the inner side (Fig. 3). The abutment was attached to the implant component which is mounted with a digital torque gauge (Tohnichi torque gauge, model BTGE50CN) and the abutment retaining screw was tightened to 30 N cm, (according to the company’s recommendation) using the digital torque gauge (Fig. 4). After 10 min, all abutment screw samples were retightened at the same torque (30 N cm) and left unloaded for 10 min. Six samples were randomly selected as the control group (group 0) to measure and record the initial RTVs of the abutment screws using the digital torque gauge. The remaining 78 samples were randomly divided into 13 experimental groups of 6 and underwent different numbers of mechanical loading cycles in the Electropuls E1000 dynamic test instrument (Instron, Fig. 5), which delivered dynamic loading forces between 15 and 250 N with a frequency of 15 Hz. The implant samples were mounted in an angled steel holder so that the axis was at a 30 ± 1° angle to the loading direction. The loading force was applied to the hemispherical metal cap of the implant samples, with no lateral constraint (Fig. 6).

**Fig. 7:**
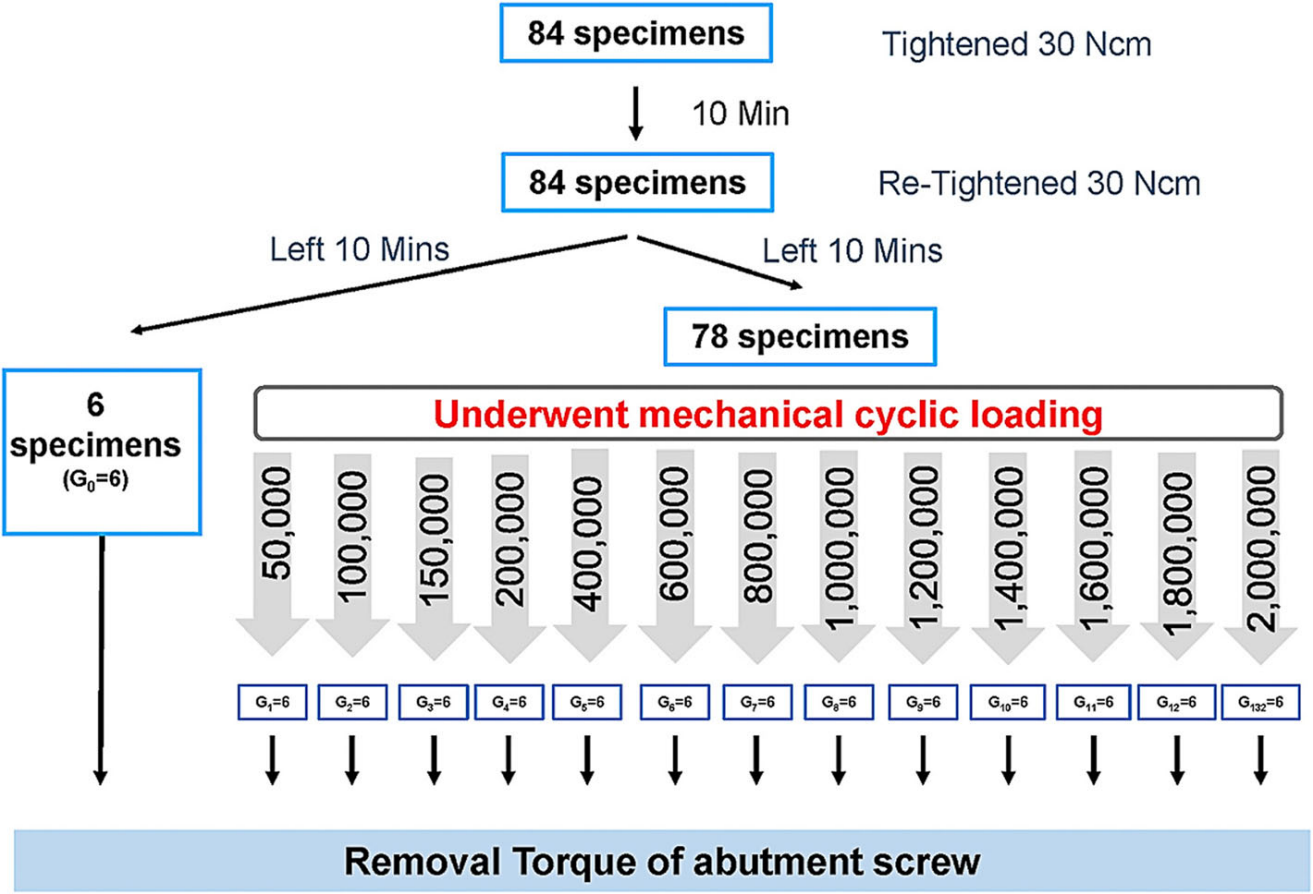
Schematic diagram for experimental procedure

The numbers of loading cycles for each experimental group were 50,000, 100,000, 150,000, 200,000, 400,000, and continually increasing 200,000 cycles per group to the last group at 2,000,000 cycles. After the cyclic loading, all implant samples were measured to determine the RTVs of the abutment screw using the digital torque gauge. The RTVs were recorded and the mean percentage loss of the RTVs in each experimental group was calculated by comparison with the initial RTVs. The schematic experimental procedures are shown in Fig. 7.

Statistical analysis was performed using one-way ANOVA for the overall effect of the numbers of mechanical loading cycles on the RTVs of the abutment screw as well as the post hoc Tukey’s HSD test SPSS 20 (IBM SPSS, Chicago, IL, USA) was used, and differences at *P* < 0.05 were considered statistically significant.

## Results

**Fig. 8:**
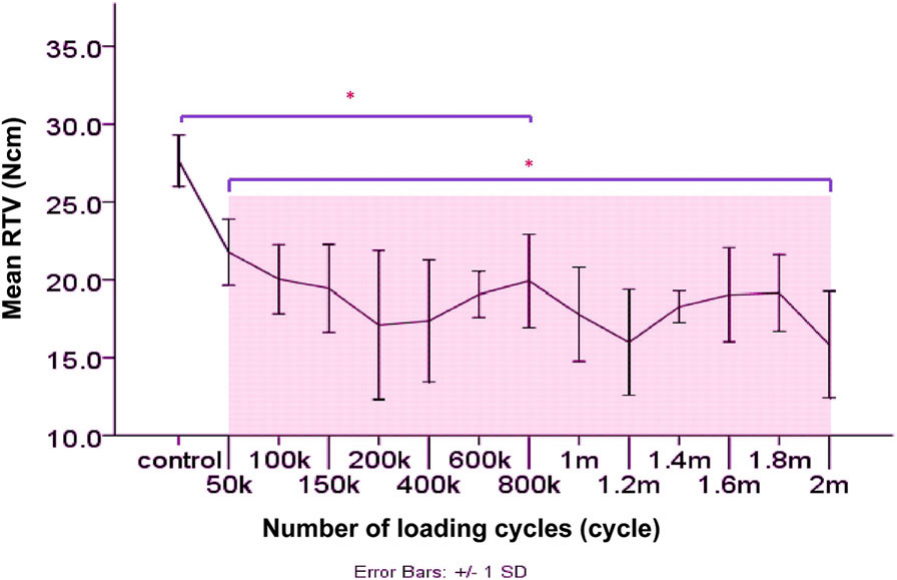
Change in RTVs according to the numbers of mechanical loading cycles

The RTV data were normally distributed (Shapiro-Wilk test). The mean RTVs (N cm) of all groups are shown in Table 1. The RTVs in all groups decreased. The mean initial RTV of the control group was 27.67 N cm, a decrease of 7.78% compared to the insertion torque. After mechanical cyclic loading, all experimental groups from 50,000 to 2,000,000 cycles of loading showed significant decreases in mean RTVs when compared with the initial RTVs (*P* < 0.05) (Table 2). Similar results were found from 50,000 cycles to 1,800,000 cycles but without significant differences (*P* > 0.05). The statistically significant reductions of RTVs were found at 2,000,000 cycles (*P* < 0.05) (Table 3, Fig. 8). The comparison of the mean RTVs among groups are shown in Table 3. Although decreases in RTVs were observed in all groups, no screw loosening occurred.

**Table 1:** Mean removal torque values (N cm) of the abutment screws of the implant-abutment connections in all groups

Group	Number of cycles	Mean removal torque value (N cm) ± SD	% of mean RTV loss compared to initial RTV
0	0	27.67 ± 1.66 (initial RTV)	–
1	50,000	21.77 ± 2.13	21.33%
2	100,000	20.03 ± 2.21	27.59%
3	150,000	19.45 ± 2.82	29.70%
4	200,000	17.10 ± 4.79	38.19%
5	400,000	17.37 ± 3.92	37.29%
6	600,000	19.07 ± 1.48	31.08%
7	800,000	19.93 ± 3.00	27.95%
8	1,000,000	17.78 ± 3.03	35.75%
9	1,200,000	15.99 ± 3.40	42.20%
10	1,400,000	18.28 ± 1.02	33.95%
11	1,600,000	19.04 ± 3.03	31.17%
12	1,800,000	19.16 ± 2.45	30.75%
13	2,000,000	15.84 ± 3.42	42.74%

**Table 2:** One-way ANOVA for the difference in removal torque values among groups with different numbers of mechanical loading cycles

		Sum of squares	df	Mean square	*F*	*p* values
RTVs	Between groups	670.997	13	51.615	6.110	.000**
	Within groups	591.291	70	8.447		
	Total	1262.287	83			

**One-way ANOVA, *p* value is considered significant at 0.05

**Table 3:** Post hoc Tukey’s HSD test of mean removal torque values in all groups

Number of cycles	Subset for alpha = 0.05			
	*N*	1	2	3
0	6	−27.6667		
50,000	6		−21.7667	
100,000	6		−20.0333	−20.0333
800,000	6		−19.9333	−19.9333
150,000	6		−19.4500	−19.4500
1,800,000	6		− 19.1583	−19.1583
600,000	6		−19.0667	−19.0667
1,600,000	6		−19.0417	−19.0417
1,400,000	6		−18.2750	−18.2750
1,000,000	6		−17.7750	−17.7750
400,000	6		−17.3667	−17.3667
200,000	6		−17.1000	−17.1000
1,200,000	6		−15.9917	−15.9917
2,000,000	6			−15.8417
*p* values		1.000	.055	.424

Means for groups in homogeneous subsets are displayed. Uses harmonic mean sample size = 6.000

**One-way ANOVA, *p* value is considered significant at 0.05

## Discussion

From the study, the RTVs of the control group, which was left unloaded for 10 min after second tightening decreased 7.78% from the tightening torque. Previous studies have shown a 2 to 10% loss of screw preload [21]. These results can be explained by the settling effect as follows [16]. Firstly, the tightening torque is used to overcome the friction of the contacting metal surface connection [32]. Secondly, wear of the contacting implant-abutment surface can cause axial displacement of the abutment into the implant bore and the length of the elongated screw is shortened microscopically, leading to the loss of screw preload [12, 33]. After mechanical cyclic loading, all experimental groups from 50,000 to 2,000,000 cycles showed significant decreases in mean RTVs compared with the initial RTVs (*P* < 0.05). These results are similar to those of several studies. Mohammed et al. [17] reported that the post-loading RTVs of internal hex implants were significantly lower than the initial RTVs after 16,000 cycles and Cibirka et al. [26] found decreasing RTVs in the internal hexagon of Nobel Biocare implants after 5 million cycles of fatigue testing. The decrease in RTVs after cyclic loading can be explained by the micromovement of the joint connection or progressive settling effect from functional loading [33]. It is assumed that mechanical cyclic loading will serve as a proxy for oral functional loading, which can cause micromovement and slipping between the abutment screw thread and the implant, reducing the tensional force and resulting in decreased preload of the screw [34, 35]. Additionally, in functionally loaded implants, the progressive settling effect and the wedge effect cause increased axial displacement of the abutment into the implant connection [36]. Seol et al. [37], who analyzed the axial displacement of an internal implant-abutment connection after cyclic loading, found that the two-piece abutment of an internal octagon connection showed continuous axial displacement, but the rate of axial displacement was slow after 100,000 cycles. In our study, the decrease in RTVs was also constant after 50,000 cycles. This might imply that the axial displacement has a great effect on the loss of screw preload. Additionally, Kim et al. [38] indicated that the RTVs of abutment screws are related to the settling value. They found that after cyclic loading, there were statistically significant differences in the settling value and also in RTVs in internal connections, whereas external connections showed no significant changes in settling values and resulted in no significant changes in RTVs. However, the relation between settling values and RTVs varies depending on many factors, such as the material, design, and characteristic of the abutment-implant interface [38].

Although the reduction in RTVs was observed after mechanical cyclic loading, no screw loosening occurred after 2,000,000 cycles of loading, which it assumed represents 3 years of function in worse scenario setting according to ISO 14801 in vivo [23]. Our study presents similar results to those of Binon and McHugh, who reported that the 30-N-cm insertion torque can maintain screw-joint stability in 3 years of simulated function [34]. The reduction of screw preload after 2,000,000 cycles of loading was 42.74%. This is considered to be relatively low and might be because of the implant-abutment connection design [39]. The eccentric force has little effect on the screw preload under functional loading because the contacting part of the cone connection helps to provide frictional resistance and mechanical stability [7, 8]. Moreover, the Octatorx lobular anti-rotational design help produce little micromovement in the joint system under load [9]. The screw design also has an effect on the screw preload [39]. Paepoemsin et al. [31] found that the retaining tapered screws of their implants maintained higher preload efficiency than did the flat head screws of the implants before and after cyclic loading (*P* < 0.05). In our study, RTVs were constant from 50,000 cycles of loading to 1,800,000 cycles. This result is not in agreement with those of Khraisat et al. [40], who concluded that 1,000,000 cycles of loading significantly affected the RTVs of CeraOne abutment external hex implants compared with 500,000 cycles of loading. This might be because different types of implant-abutment connections were used. In external connections, implant-abutment stability is obtained primarily by the tension of the screws [4]. Therefore, the screw preload in external connections is affected by the cyclic loading more than in internal connections.

Cho et al. [39] studied the effect of retightening the abutment screw on RTVs in internal connection implants under cyclic loading at 3, 10,100, and every 20,000 cycles up to 100,000 cycles. They found that most of the decrease in RTVs occurred at 10 cycles, and after that, RTVs did not change significantly. The study showed that retightening the abutment screw under cyclic loading resulted in superior RTVs when compared with no retightening [39]. However, retightening the abutment screw might change the shape of the abutment screw and the inner screw thread of the implant [41]. In our study, we demonstrated decreasing the RTVs after cyclic loading without retightening the abutment screw.

Tzenakis et al. [42] reported that retightening of the screw is strongly recommended, but the appropriate timing of retightening is still not clear. Cho et al. [39] recommended retightening abutment screws in the early stage of functional loading, at the first week in vivo, in internal type implants because they found no significant difference in RTVs between 20,000 and 100,000 cycles of loading. In our study, the RTVs decreased in the early stage at 50,000 cycles of loading and then remained constant to 1,800,000 cycles. The result can be used to estimate that the implant should also be retightened in the early stage of cyclic loading under a worst-case situation [26]. Seol et al. [37] stated that to minimize screw loosening, the screw should be retightening at 1 month in vivo after constant axial displacement at 100,000 cycles of loading. Nevertheless, different implant-abutment connections, designs, and materials influence the screw preload differently. Therefore, it may be summarized that re-tightening is a theoretical recommendation based on in vitro studies that have found it beneficial in terms of RTV reduction.

This study cannot compare the RTVs to the other implant-abutment connections because each implant system has their own manufacturing technique especially the tolerance value between implant and abutment connection, and these values are considered as the confidential data of the company. However, by conforming to the international standard of the medical device for dental implant systems (ISO 13485), the results of this study may be applicable to other combined mandatory index-cone connections of other commercial implant systems. Clinically, the retightening of the abutment screw is recommended after the first 6 months in function because the significant preload loss at the early cyclic loading as shown in our and other studies [39]. The 6-month or yearly regular clinical follow-up after dental implant treatment is suggested. In the clinical situation, the screw loosening leads to implant prosthesis movement and in the worst case, the abutment screw may break. The RTV value which related to the screw and implant-abutment connection loosening in the clinical situation is still not clear and further clinical studies are required.

In addition, further studies are suggested to examine the relation between the settling value [38] and the preload value of the screw. It would be helpful for evaluating the stability of the implant-abutment connection after functional loading. The decreasing pattern of RTVs after cyclic loading was specific to the design of the implant-abutment connection, so more studies are recommended in other implant systems.

## Conclusion

According to the conditions for the fatigue test set out in ISO 14801:2007, the RTVs of the combined cone and octalobule index reduced significantly after 50,000 cycles and did not change significantly until 2,000,000 cycles.

## Abbreviations

RTVs: Removal torque values
